# Ultrasonographic Features of Kidney Transplants and Their Complications: An Imaging Review

**DOI:** 10.5402/2013/480862

**Published:** 2012-12-02

**Authors:** Chrysafoula Kolofousi, Konstantinos Stefanidis, Demosthenes D. Cokkinos, Dimitrios Karakitsos, Eleni Antypa, Ploutarhos Piperopoulos

**Affiliations:** ^1^Radiology Department, Evangelismos Hospital, 10676 Athens, Greece; ^2^Intensive Care Unit, General State Hospital of Athens, 11527 Athens, Greece

## Abstract

Renal transplantation is the treatment of choice for managing patients with end-stage kidney disease. Being submitted to a very serious surgical procedure, renal transplant recipients can only benefit from follow-up imaging and monitoring strategies. Ultrasound is considered as the principal imaging test in the evaluation of renal transplants. It is an easily applied bedside examination that can detect possible complications and guide further imaging or intervention. In this imaging review, we present essential information regarding the sonographic features of healthy renal transplants, detailing the surgical technique and how it affects the sonoanatomy. We focus on various complications that occur following renal transplantation and their sonographic features by reviewing pertinent literature sources and our own extensive imaging archives.

## 1. Introduction

Renal transplantation is considered a treatment of choice for end-stage kidney disease (ESKD) since the 1960s. It is cost-effective and provides better long-term survival and better life quality in comparison to hemodialysis and/or peritoneal dialysis [1].

With improved transplantation technology, new generations of immunosuppressive agents and developments in graft preservation techniques, the 1-year survival rates for grafts, are reported to be 80% for mismatched cadaveric renal grafts; 90% for nonidentical living related grafts; 95% for human lymphocyte antigen-identical grafts. The half-life of grafts from living related donors varies between 13 and 24 years, depending on the match [2].

Two-dimensional ultrasound (US) was introduced in the evaluation of renal transplants back in the 1970s, while the application of Doppler techniques in the following years established the method in routine practice. US is a relatively cheap, noninvasive, and nonnephrotoxic modality, which may be applied for diagnostic and monitoring purposes early on, in the posttransplant period, establishing thus a baseline for follow-up scanning. Its role in the evaluation of early graft complications is of paramount importance as besides detecting vascular pathology (i.e., arterial stenosis and venous thrombosis); it can be also utilized for guided renal biopsy and drainage of fluid collection [3–5]. Hereby, we present basic points of US evaluation in kidney transplantation.

## 2. Ultrasound Evaluation of the Healthy Renal Transplant

**Figure 1:**
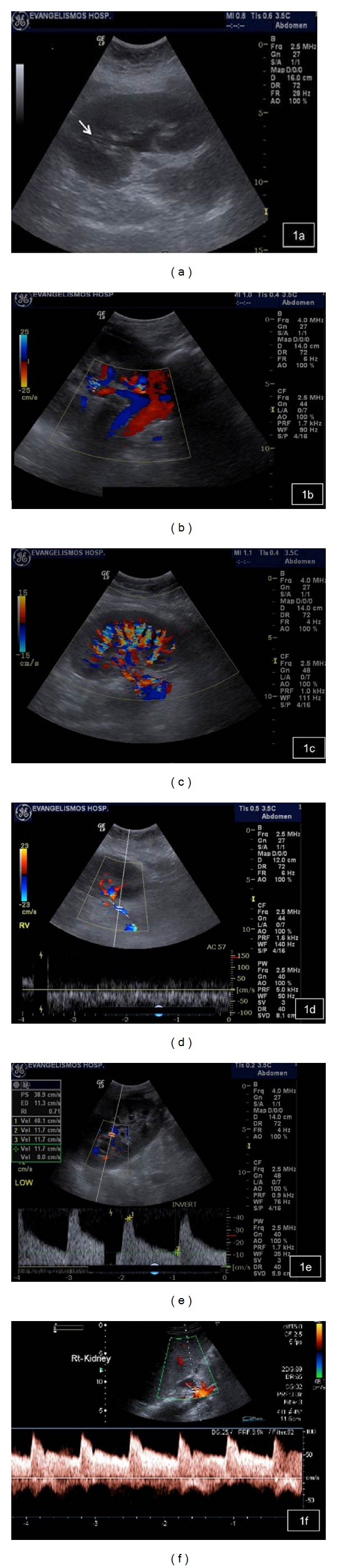
(a) Normal transplant kidney on gray scale ultrasound, demonstrating good contrast resolution between cortex and medulla. Slight dilatation of the collecting system and pig-tail catheter (arrow) is noted. (b) Normal renal artery and vein of the transplant kidney on color Doppler ultrasound. (c) Normal homogeneous blood flow throughout the transplant kidney on color Doppler ultrasound. Interlobar, arcuate, and the peripheral cortical branches are illustrated. (d) Normal renal vein waveform on spectral Doppler ultrasound. (e) Normal intrarenal artery waveform on spectral Doppler ultrasound shows a brisk systolic upstroke and high diastolic flow. Resistive index is normal (RI = 0.71). (f) Normal waveform of the renal artery on spectral Doppler ultrasound.

A baseline US evaluation is performed in the first 24–48 hours posttransplantation. A detailed examination protocol includes renal size and echogenicity, collecting system and ureter condition and evaluation of any postoperative collections. Color and spectral Doppler imaging should assess flow in the renal and iliac vessels, flow velocity measurements, as well as evaluation of the intrarenal vessels. “Flow quantification” can be measured by the resistivity index (RI), pulsatility index (PI), and systolic/diastolic ratio. An example of sonographic evaluation of a renal transplant in gray scale and color and spectral Doppler is given in Figure 1. The first baseline US examination identifies any early complications that may need to be attended urgently to secure the graft.

The healthy transplanted kidney has comparable ultrasound appearance to the healthy native one; however, a more detailed two-dimensional image is apparent as the transplant is usually located more superficially, and thus higher-frequency transducers can be utilized [5]. The reniform outline and central echo complex, resulting from the collecting system and the renal vasculature together with any sinus fat, are well depicted, while distinction between the renal cortex and the relative echo-poor medulla is usually apparent [4] (Figure 1(a)). Transplant sizes are similar to native kidneys; however, gradual increase of its dimensions can be seen over the first few weeks by up to 32% of the initial length by the fourth week [6]. The collecting system of a well-functioning transplant is often slightly dilated, presumably because of a combination of an increased volume of urine produced (because it is acting as the sole kidney) and loss of the ureter's tonicity from denervation (Figure 1(a)). However, in the unobstructed transplant, the filling should be minor and confined to the renal pelvis, while filling of the infundibula or the calyces is suspicious of significant outflow obstruction.

The vessels of the healthy transplant are usually better demonstrated than those of the native kidney, such that color flow is expected to fill out to the renal capsule when using power Doppler, at least in the midportion of the kidney closest to the transducer [7] (Figures 1(b) and 1(c)). Spectral Doppler signals from the segmental and interlobar vessels show the normal fast systolic upstroke with a subsequent slow decay in diastole, forward flow being maintained until the next cardiac cycle. Thus, RI values of 0.8 or lower are expected, although the clinical context should be evaluated as well (Figure 1(e)). The main renal artery is usually readily demonstrated but is often much more tortuous than that of the native kidneys because of the way the transplant is relocated into the iliac fossa after the anastomoses have been performed. Therefore, optimum locations for Doppler measurements, the straight portions of the artery, are those that tend to lie parallel to the skin and thus subtend the worst beam-to-vessel angles (Figure 1(f)). The branch and main renal veins are easily accessed for Doppler studies [4] (Figure 1(d)).

## 3. Surgical Technique

Knowledge of the basic surgical procedures and of the postsurgical anatomical features is required for the detailed sonographic evaluation of the renal transplant. The transplant kidney is placed extraperitoneally in the right or (less commonly) left iliac fossa [8]. Vascular anastomoses are made with the external iliac vessels. In cadaveric transplants, the donor renal artery is obtained with a small aortic patch (“Carrel patch”) and is anastomosed end-to-side with the recipient external iliac artery. If multiple renal arteries exist in the donor kidney, a long “Carrel patch” containing all renal arteries or separate patches is obtained. In living donors, the main renal artery is obtained with the kidney and is anastomosed either end-to-side to the external iliac artery or end-to-end to the internal iliac artery. The renal vein is anastomosed end-to-side to the external iliac vein. When multiple renal veins exist, the larger is anastomosed and the smaller are ligated.

The ureter is anastomosed by creating a new ureterocystostomy. The donor ureter is tunneled through the bladder wall along the dome, resulting in a new ureteral orifice, higher than the native orifices [8]. Less commonly, an ureteroureterostomy or a pyeloureterostomy can be created. In pediatric recipients of adult donors, kidneys may be placed in a more cephalad abdominal position and anastomosed to the distal aorta and inferior vena cava. In pediatric cadaveric donors, both kidneys with the aorta and inferior vena cava may be harvested and transplanted in a single adult recipient.

## 4. Complications

US exhibits pivotal role in the monitoring of renal grafts, in the detection, management, and followup of both early and late complications. Posttransplant complications can be divided into parenchymal, vascular, and collecting system abnormalities, perinephric fluid collections, neoplasms, and recurrent native renal disease.

### 4.1. Parenchymal Abnormalities

Diseases of the renal parenchyma are usually diffuse, leading to graft dysfunction. They include acute tubular necrosis (ATN), hyperacute, acute and chronic rejection, drug nephrotoxicity, and infection. Differential diagnosis is difficult by imaging alone, and US is not sensitive or specific in this task [1]. Distinction still relies on biopsy [4]. Absent end-diastolic flow within the first week posttransplantation, although associated with impaired renal functional recovery, failed to be of prognostic value in long-term graft function and/or survival [9]. Despite the fact that US has not proved to be as accurate in the evaluation of parenchymal dysfunction as initially thought it still has a central role in the qualitative assessment of graft perfusion. The latter, in relation with clinical and biochemical findings, can assist in monitoring any emerging graft dysfunction and in guiding biopsy, when needed [10].

#### 4.1.1. Acute Tubular Necrosis

**Figure 2:**
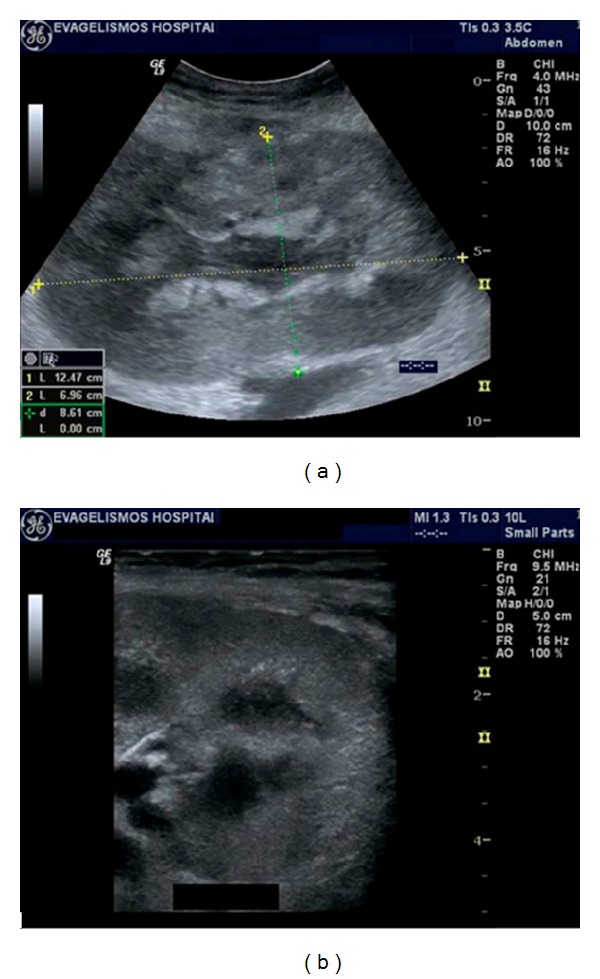
(a,b) Acute tubular necrosis (ATN) of 2 cadaveric renal transplants a few days after transplantation. Gray scale ultrasound demonstrates edematous appearance and loss of normal cortical medullary differentiation in both transplanted kidneys.

Acute tubular necrosis (ATN) is a common cause of early posttransplantation renal function impairment. More common in cadaveric donors than in living related donors, it usually resolves in 2 weeks. It is caused by donor kidney ischemia during transplantation and reperfusion injury [11]. US is normal or only reveals nonspecific findings such as renal enlargement, altered echogenicity of parenchyma and pyramids, and reduced diastolic flow (elevated RI and PI Doppler indices) in the interlobar vessels (Figure 2). Sometimes, this can be so marked that end-diastolic flow can be completely absent or even reversed [12]. However, this finding is not pathognomonic for ATN as it can also be observed in severe rejection and renal vein thrombosis [10]. Nevertheless, ATN is usually well controlled by the newer immunosuppressive drugs.

#### 4.1.2. Rejection

Rejection, depending on the time of occurrence, is classified into hyperacute, acute, or chronic. Hyperacute rejection is rare, caused by preformed antibodies in the recipient's serum. It occurs in the operating room, immediately postsurgery [1]. As a result, these cases are rarely imaged. 

#### 4.1.3. Acute Rejection

**Figure 3:**
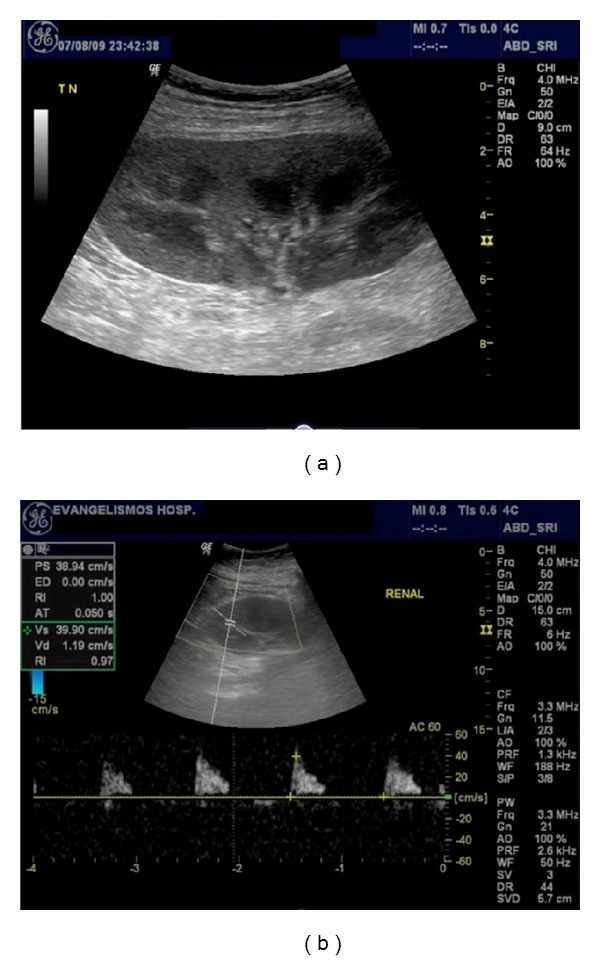
Acute rejection. On gray scale ultrasonography, the kidney is mildly swollen with compression of its sinus fat. Spectral Doppler waveforms of intrarenal arteries are monophasic with loss of diastolic flow and increased resistivity index (RI = 1).

Acute rejection is the most common type of rejection (10%–37%), usually occurring 1–3 weeks after transplantation [13]. Despite effective management with high-dose steroids and immunosuppressive medication, an episode of rejection is an adverse long-term prognostic indicator [14]. The patient is often asymptomatic, but flu-like symptoms, pyrexia, and graft tenderness may be present. On US, associated two-dimensional and Doppler features have been shown to be nonspecific. Kidney enlargement, hyper- or hypoechogenicity, or even normal appearance is possible. Doppler may reveal high PI and RI values (>0.9). In very serious cases, renal artery that reversed diastolic flow may be seen (Figure 3). These findings are similar to those of ATN, and these two entities can be differentiated by the time course of the finding. Acute rejection rarely develops in the first few days after transplantation [5].

#### 4.1.4. Chronic Rejection

Chronic rejection or chronic allograft nephropathy (CAN) is the most common cause of late graft failure, beginning at least 3 months after transplantation [13]. Renal function progressively deteriorates and eventually fails. Many factors may contribute to the pathogenesis of CAN such as subclinical rejection, ATN, drug toxicity, and donor age. However, the main predisposing factor is previous episodes of acute rejection [14]. Therefore, efforts to prevent episodes of acute rejection can be an effective method of reducing chronic rejection. The diagnosis is made histologically by demonstrating an overall fibrotic picture affecting the vascular endothelium, tubules, glomeruli, and interstitium [15]. US appearance is not typical, ranging from normal to hyperechogenic, along with cortical thinning, reduced number of intrarenal vessels, and mild hydronephrosis.

#### 4.1.5. Drug Toxicity

The calcineurin inhibitors cyclosporine and tacrolimus are key immunosuppressive agents administered to avoid acute rejection. Unfortunately, they are potentially nephrotoxic, causing vasoconstriction on the afferent glomerular arterioles and with long-term use interstitial fibrosis [10]. Recently, a polyoma virus infection (the BK virus) has been described and believed to be a latent virus that may become reactivated in immunosuppressed patients and cause a nephropathy indistinguishable from rejection (or ATN) [16]. US can be either normal or nonspecific (increased RI values may be found on Doppler examination). Findings should be related with the serum drug levels. In the short term, nephrotoxicity from cyclosporine is dose depended and responds to a reduction in dosage [15].

#### 4.1.6. Infection

More than 80% of renal transplant recipients suffer at least one case of infection during the first year after transplantation. Especially in the first 6 months, patients are at increased risk of opportunistic infections, and immunosuppressive medication, indwelling catheters, and frequent glycosuria are contributing risk factors. Early diagnosis of infections and prompt administration of therapy may help prevent loss of graft function and improve patient outcome [17].

**Figure 4:**
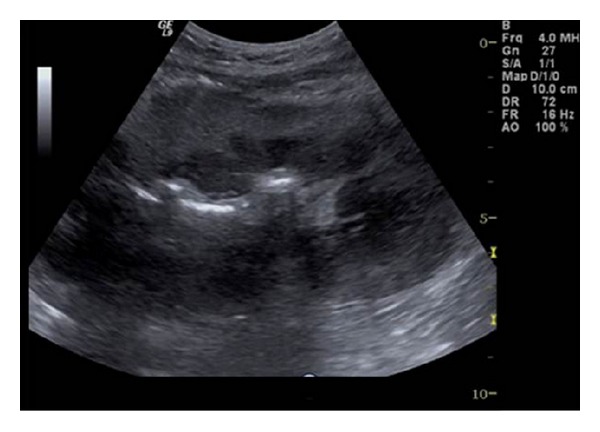
Emphysematous pyelonephritis. Ultrasound image demonstrates mildly increased cortical echogenicity and gas in the parenchyma of the renal graft, which produces echogenic lines with distal reverberation artifacts.

Patients may present with fever of unknown origin, pain, or be asymptomatic due to their immunosuppressed state, which in turn may cover the clinical features of a pyelonephritis [18]. As with the native kidney, the sonographic appearance of transplant infections is quite variable and nonspecific. Urothelial thickening and focal or diffuse areas of increased or decreased echogenicity are recognized findings, although these features can be present in the early stages of rejection. Any echogenicity within a dilated pyelocaliceal system is usually clinically significant and suggestive of pyonephrosis, while focal rounded, weakly shadowing, and echogenic structures within the collecting system are fairly specific for fungus balls. In emphysematous pyelonephritis, gas in the parenchyma of the renal graft produces an echogenic line with distal reverberation artifacts (Figure 4). Papillary necrosis can also be the result of certain infections and may subsequently lead to ureteric obstruction but with no typical sonographic findings. Finally, abscesses have a complex, cystic, nonspecific appearance on US and may be treated with either US- or CT-guided percutaneous drainage. The latter usually respond well to external drainage and systemic antibiotics [17].

#### 4.1.7. Failed Transplants

Nonfunctional renal allografts are often left in situ, while patients revert to chronic dialysis therapy or undergo retransplantation. Usually, their size decreases and may reveal extensive cystic changes (especially after long-term dialysis), fatty replacement hydronephrosis, infarcts, hemorrhage, and calcifications. Therefore, failed renal transplants must be differentiated from pelvic tumors and, in cases of dense calcifications, from contrast material-filled bowel on computed tomography (CT) [19].

### 4.2. Vascular Complications

Vascular complications occur in fewer than 10% of renal transplant recipients but are an important cause of graft dysfunction with high associated morbidity and mortality. In contrast to other causes of transplant dysfunction, once identified, vascular lesions are usually easily repaired by radiological intervention.

Despite the fact that magnetic resonance (MR) angiography is superior in the diagnosis of vascular complications, color Doppler US although conventional remains an excellent noninvasive technique for evaluating vascular pathology [5, 10]. Knowledge of the surgical anatomy is a prerequisite for correct interpretation of the findings.

#### 4.2.1. Renal Artery Stenosis

Transplant artery stenosis is the most common vascular complication (up to 10%) [3, 20–22]. It usually occurs within the first 3 months [20]. Strictures can affect the iliac artery just proximal to the anastomotic site (atherosclerotic disease in the donor vessel, surgical clamping injury), the anastomosis itself (related to surgical technique), or the proximal renal artery (intimal ischemia). Approximately half of renal artery stenosis can be located adjacent to the anastomosis; moreover, end-to-end anastomoses have a threefold greater risk of stenosis than end-to-side anastomoses [23].

Evaluation for renal artery patency should be performed in several clinical scenarios: (a) severe hypertension refractory to medical therapy, (b) hypertension combined with an audible bruit over the graft, and (c) hypertension associated with unexplained graft dysfunction (3). Moderate hypertension alone is not a precise marker for renal artery stenosis because up to 65% of transplant recipients have nonrenovascular hypertension.

**Figure 5:**
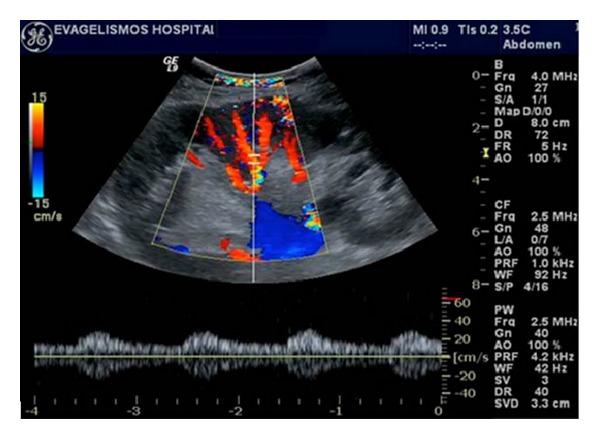
Spectral Doppler ultrasound image shows a tardus parvus waveform in an intrarenal arterial branch, distal to renal artery stenosis.

The renal artery is mapped by using color Doppler techniques. The stenotic segments reveal focal color aliasing due to increased flow velocity. Doppler criteria for significant stenosis include the following: (a) velocities greater than 200 cm/s or a focal frequency shift greater than 7.5 KHz (when a 3-MHz transducer is used), (b) a velocity gradient between stenotic and prestenotic segments of more than 2 : 1, and (c) marked distal turbulence (spectral broadening). In the segmental branches of the transplant, tardus-parvus waveform abnormalities may variably be observed (Figure 5). The latter is often seen as an indirect sign of a significant proximal arterial stenosis. The Doppler indices used to define this waveform include prolonged acceleration time (>0.07 s); diminished acceleration index (<300 cm/s^2^); decreased RI (<0.56); loss of early systolic peak [22, 24–26]. The combination of both direct and indirect Doppler measurements gives an overall accuracy of 95% for detecting renal artery stenosis [27].

Even if these findings exist, when the patient is clinically doing well, only conservative monitoring is performed [24]. When treatment is necessary, percutaneous transluminal angioplasty with or without stent placement is nowadays accepted as the initial treatment of choice [28]. Clinical success in the form of improvement or definite treatment has been reported in 73% of patients.

#### 4.2.2. Infarction

Thrombosis of the main renal artery occurs very rarely (<1% of cases) in the early postoperative period and usually leads to graft loss. It may result from severe rejection, anastomotic occlusion, arterial kinking, or intimal flap. Patients with renal transplant infarction present with anuria and often with swelling and tenderness over the graft [17].

**Figure 6:**
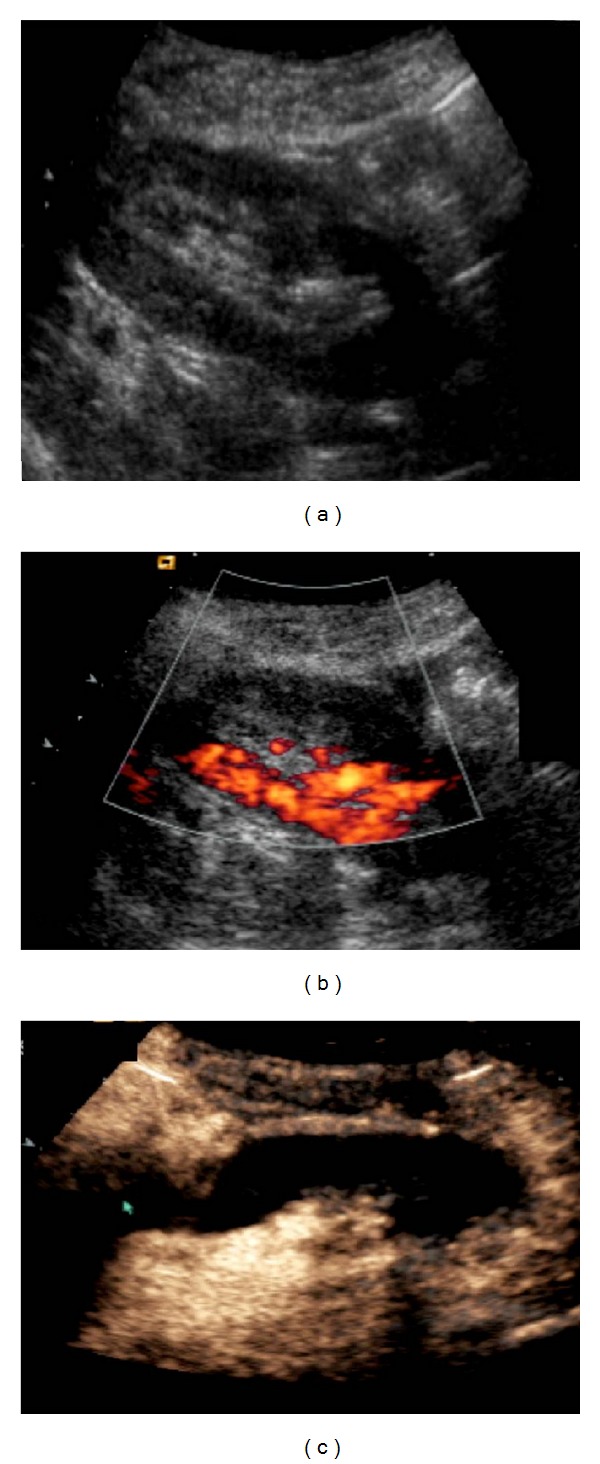
Transplant kidney infarct. It is not evident on B-mode ultrasound (a). Power Doppler imaging reveals a large blood flow defect in the largest part of the kidney (b). Perfusion absence is confirmed by contrast enhanced ultrasound with contrast agent (arrow) (c).

In cases of global infarction the kidney appears hypoechoic and may be diffusely enlarged on US examination. Color-Doppler imaging reveals no arterial and venous flow distal to the thrombus and in the intrarenal vessels. Similar findings can be present at severe rejection. Therefore, angiography or MR angiography may be performed for further investigation. Thrombosis of an accessory renal artery or intrarenal arterial branches will result in segmental infarcts. On US, a segmental infarct produces a focal, hypoechoic, typically wedge-shaped area with perfusion defects on color US and postinjection of contrast agents (Figure 6). However, these findings may also be seen in severe pyelonephritis or transplant rupture.

Although a main artery thrombosis usually results in nephrectomy, there has been some reported success with percutaneous angiographic thrombolytic techniques for treating infarcts. Early diagnosis and treatment are vital for allograft salvage [29].

#### 4.2.3. Renal Vein Thrombosis

renal vein Thrombosis is an unusual posttransplant complication; it happens in <5% of patients within the first postoperative week. Clinical presentation is similar to infarction with abrupt cessation of urinary function, swelling, and tenderness over the graft. Renal vein thrombosis is more likely to occur following surgical difficulty with the venous anastomosis, episodes of hypovolemia, venous compression by a peritransplant collection, or slow flow secondary to rejection. An increased predominance of renal vein thrombosis in the left lower quadrant allografts has also been attributed to compression of the left common iliac vein between the sacrum and the left common iliac artery (silent iliac artery compression syndrome) [23].

On US, the kidney may be large and hypoechoic with loss of corticomedullary differentiation. Echogenic material may be seen in the renal vein. Doppler examination shows reduced or no flow in the main renal vein, and there is increased resistance on the arterial conduit, often resulting in reversed diastolic flow in the main renal artery and/or intrarenal arteries [30–32]. If thrombosis is partial, high RI may be seen [1]. Increased focal venous velocity may also be noted in partial thrombosis, kinking, and extrinsic pressure by fluid collection.

Diastolic flow reversal can sometimes be seen in ATN or acute rejection. However, the combination of this finding with absence of venous flow at the hilum is diagnostic for this condition and early recognition of this pattern is crucial because the allograft may sometimes be salvaged by prompt thrombectomy.

#### 4.2.4. Arteriovenous Fistulas and Pseudoaneurysms

**Figure 7:**
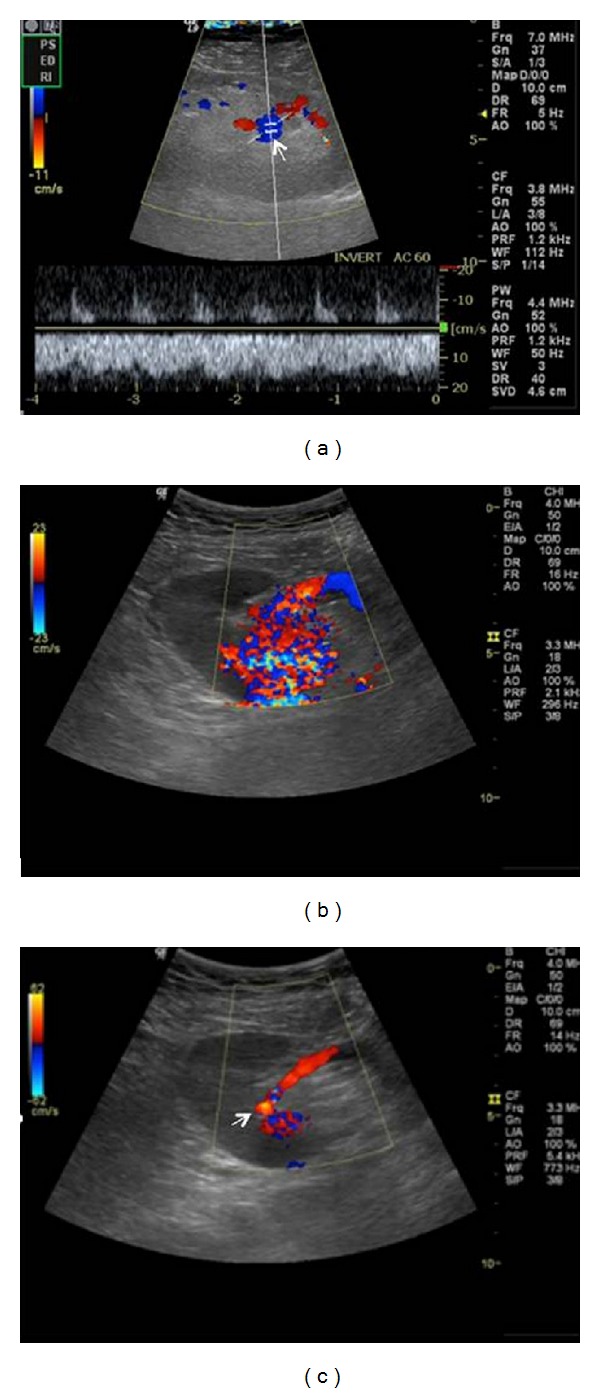
Intrarenal arteriovenous fistula following biopsy. Color Doppler ultrasound demonstrates a highly vascular lesion (arrow) with aliasing. Spectral Doppler image shows the characteristic mixed arterial venous waveform, with high velocities and low impedence.

**Figure 8:**
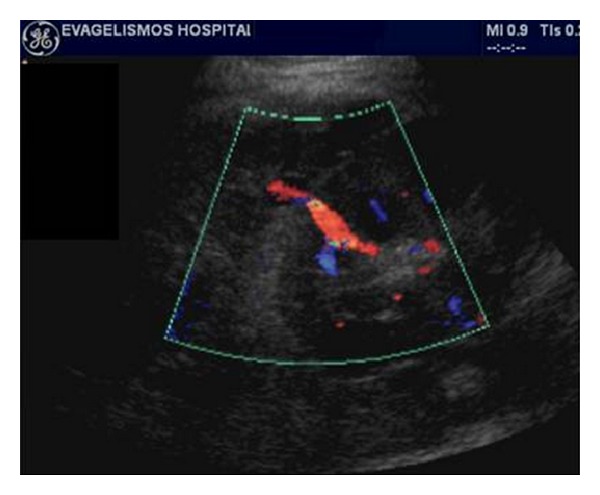
Arteriovenous fistula. Color Doppler ultrasound shows an abnormal focus of increased turbulent flow in the midpole.

Arteriovenous Fistulas (AVFs) are well-recognized complications of renal biopsies (1%–16% of biopsies), usually following a self-limiting course and resolving spontaneously [33]. AVFs form when the biopsy needle strikes both arterial and venous walls. Color-Doppler reveals an area of turbulent flow and aliasing, with very high velocity and low RI of feeding artery as well as “arterialized” flow of draining vein [11] (Figures 7 and 8). AVFs have no hemodynamic consequence and are simply observed, but occasionally they can bleed or increase in size and result in renal ischemia due to “steal phenomenon” requiring radiological embolization.

A pseudoaneurysm (PA) is a rare complication (6% of biopsies) and is due to arterial wall injury from the biopsy needle. It appears as a cystic structure on US with turbulent, swirling flow, whereas a characteristic to-and-fro waveform may be seen at the neck of the PA on spectral Doppler. Most of them thrombose spontaneously, but if there is a significant increase in size (>2 cm) transcatheter embolization should be considered. An extrarenal PA is very rare, usually occurring at the site of arterial anastomosis due to surgical technique or infection. It is accompanied with high mortality rate if ruptured [34].

### 4.3. Collecting System Complications

About two-thirds of early urologic complications (urine leaks and obstruction) appear in the first 30 days posttransplantation. In contrast to the high mortality rates of older reconstructive techniques for the restoration of urinary tract continuity (ureteroureterostomy or pyeloureterostomy) currently patients undergo ureteroneocystostomy and have a lower incidence of urologic complications (1%–8%) with very low patient mortality [17, 35].

#### 4.3.1. Urine Leaks and Urinomas

Extravasation of urine may occur from the renal pelvis, ureter, or ureteroneocystostomy site due to the surgical technique or ureteral ischemia and necrosis. Urinomas vary in size and usually appear in the first 2 weeks after transplantation between the renal graft and the bladder. Patients with renal leakage may present decreased urine output and manifest pain, tenderness around the graft, discharge from the wound, or even ipsilateral leg swelling, scrotal, or labial edema.

**Figure 9:**
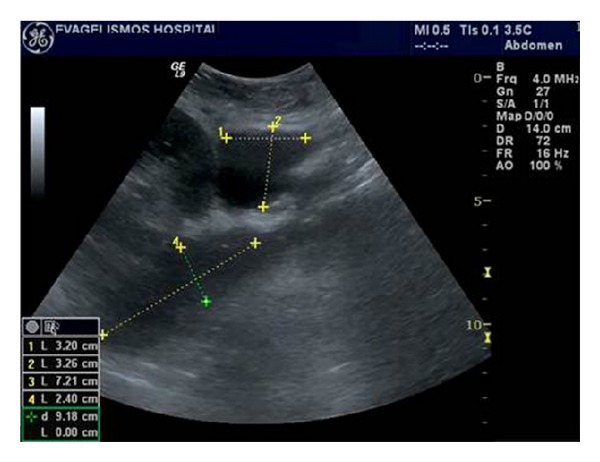
Gray scale sonogram shows two anechoic areas, without septations, next to a renal transplant. Ultrasonographically guided aspiration revealed increased levels of creatinine, compatible with urinomas.

On US, a urine leak or urinoma appears as an anechoic fluid collection with well-defined borders and lack of septations (Figure 9). Its size increases rapidly, and often drainage needs to be performed with ultrasound guidance to relieve compression and urinary ascites. The higher creatinine level of the fluid compared with its serum concentration differentiates a urine leak from seroma or lymphocele [17]. In addition, urinomas can become infected and eventually form abscesses. Antegrade pyelography is necessary to depict the site of leak and to plan the appropriate intervention. Small urine leaks may be treated with percutaneous nephrostomy and stent placement.

#### 4.3.2. Urinary Obstruction

**Figure 10:**
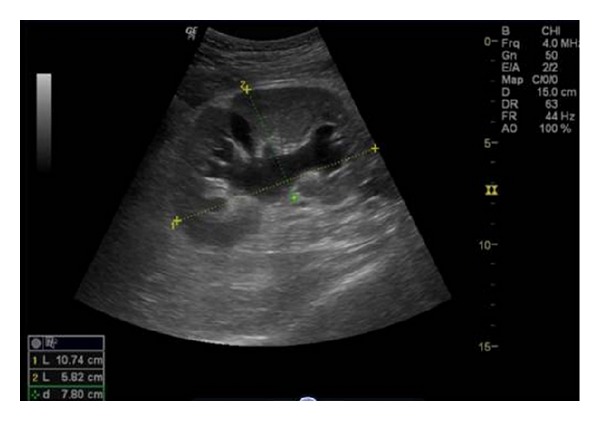
Gray scale ultrasound shows mild hydronephrosis of a renal allograft secondary to ureteral stricture.

**Figure 11:**
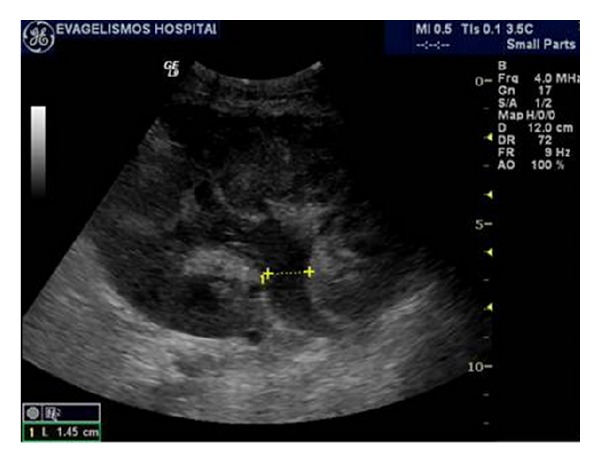
Gray scale ultrasound of a well-functioning renal transplant demonstrating slight dilatation of its collecting system. The increased volume of urine produced and the loss of ureter's tonicity, due to denervation, contribute to mild benign pelvicalyceal dilatation.

Urinary obstruction is found in approximately 2% of cases. More than 90% of ureteral stenosis occur within the distal third of the ureter, reflecting its relatively poor blood supply. Strictures are usually observed at the ureterovesical junction and may be due to scarring secondary to ischemia or rejection, surgical technique, or kinking. Less commonly, peritransplant fluid collections may compress the ureter, whereas pelvic fibrosis, calculi, papillary necrosis, fungus balls, and clots apart rare causes of urinary obstruction [36]. Due to kidney and ureter denervation, there is no typical renal colic [1]. Urinary obstruction manifests by a rising level of serum creatinine, whereas US can easily confirm the diagnosis of hydronephrosis (Figure 10). The differential diagnosis from chronic rejection may be difficult since both cause elevated titles of serum creatinine. In addition, mild dilatation of the collecting system may occasionally be seen in cases of chronic rejection [32]. Minor collecting system dilatation can be a normal finding in the early transplant kidney, due to tonicity loss secondary to denervation and increased flow through the single functioning kidney (Figure 11). The evaluation of any moderate degree of collecting system dilatation should be made in the presence of an empty bladder, as a distended bladder alone can be the underline cause. Internal echoes in the collecting system suggest pyonephrosis, fungal infections, clots, or tumor [1]. US also shows lymphoceles, hematomas, abscesses, and urinomas that may cause ureteral compression.

Percutaneous nephrostomy is usually the early treatment of choice to relieve obstruction and allow the deployment of other interventional procedures, such as ureteral stent placement and balloon urethroplasty. US-guided drainage of fluid collections is often preferred to correct the extrinsic compression they exert on the collecting system. Surgical reconstruction may be required for long or recurrent strictures [37].

#### 4.3.3. Calculous Disease

**Figure 12:**
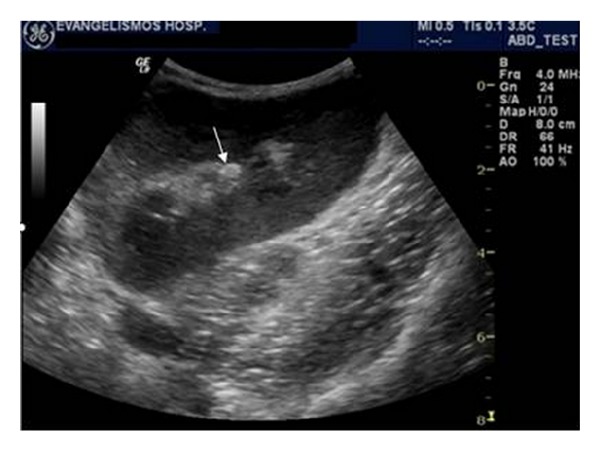
Renal transplant calculus. Ultrasonographic image of a renal graft demonstrating a shadowing echogenic focus located in the middle calyceal group (arrow). There are no obstructive effects of the collecting system of the kidney.

Compared with the general population, renal transplantation recipients are at increased risk for development of urinary calculi, with 1% to 2% developing clinically relevant stones [9]. Persisting secondary hyperparathyroidism has been reported in a significant number of patients after transplantation, whereas in the first postoperative year, 15% of patients may be hypercalcemic, which increases the risk of renal stone formation [38]. As the kidney is denervated, the patient will not suffer typical renal colic; thus an acute deterioration in renal graft function may raise the suspicion of urinary calculi. Ultrasonography reveals the same findings as in the native kidney, with a strongly reflective focus of variable size producing acoustic shadowing and twinkling artifact on color-Doppler, especially helpful in confirming small ureteric stones (Figure 12). Percutaneous nephrostomy is valuable because it decompresses the pyelocaliceal dilated system and stabilizes renal function. Most stones can be removed with endoscopic techniques.

### 4.4. Perinephric Fluid Collections

Perinephric fluid collections are observed in half of transplant recipients and include hematomas, lymphoceles, urinomas, and abscesses. The clinical relevance of these collections is largely determined by their size, location, and possible growth. Peritransplant fluid collections can be partially differentiated according to the time interval after transplantation. Small hematomas, seromas, and urinomas are usually expected in the immediate postoperative period. Lymphoceles generally occur 4 to 8 weeks after the surgical operation. Furthermore, growing collections may represent urine leaks, abscesses, or vascular injury [17]. The US features of perinephric fluid collections are nonspecific and percutaneous aspiration is the only safe way to diagnosis [39].

#### 4.4.1. Hematomas

Hematomas are relatively common in the immediate posttransplant period but may also develop spontaneously or after trauma or injury. They are usually located within the subcutaneous tissues or around the transplant, and most of them resolve spontaneously. However, large hematomas can displace the graft and produce hydronephrosis or compromise the vascular supply [17, 39].

**Figure 13:**
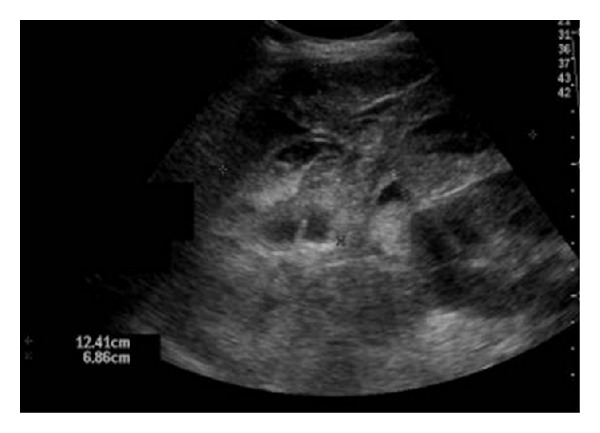
Hematoma. Longitudinal ultrasound image demonstrating a complex echogenic mass in contact with the upper pole of the transplanted kidney. There were no clinical signs of renal dysfunction, in the previous days, except from pain in the area due to an incidental injury of the recipient.

On US, acute hematomas appear complex and echogenic. With time they become more defined and cystic and often develop fibrinous septations and clot debris (Figure 13). The dimensions of any such collection should be measured on the baseline US scan because any increase in size may indicate surgical intervention. More complex collections documented later in the postoperative period with clinical evidence of infection may represent abscesses [3].

#### 4.4.2. Lymphoceles

Lymphoceles are the most usual peritransplant fluid collections affecting up to 20% of the patients [5]. They usually occur 1-2 months postsurgery owing to the surgical disruption of the lymphatic channels along the iliac vessels or around the hilum of the graft.

**Figure 14:**
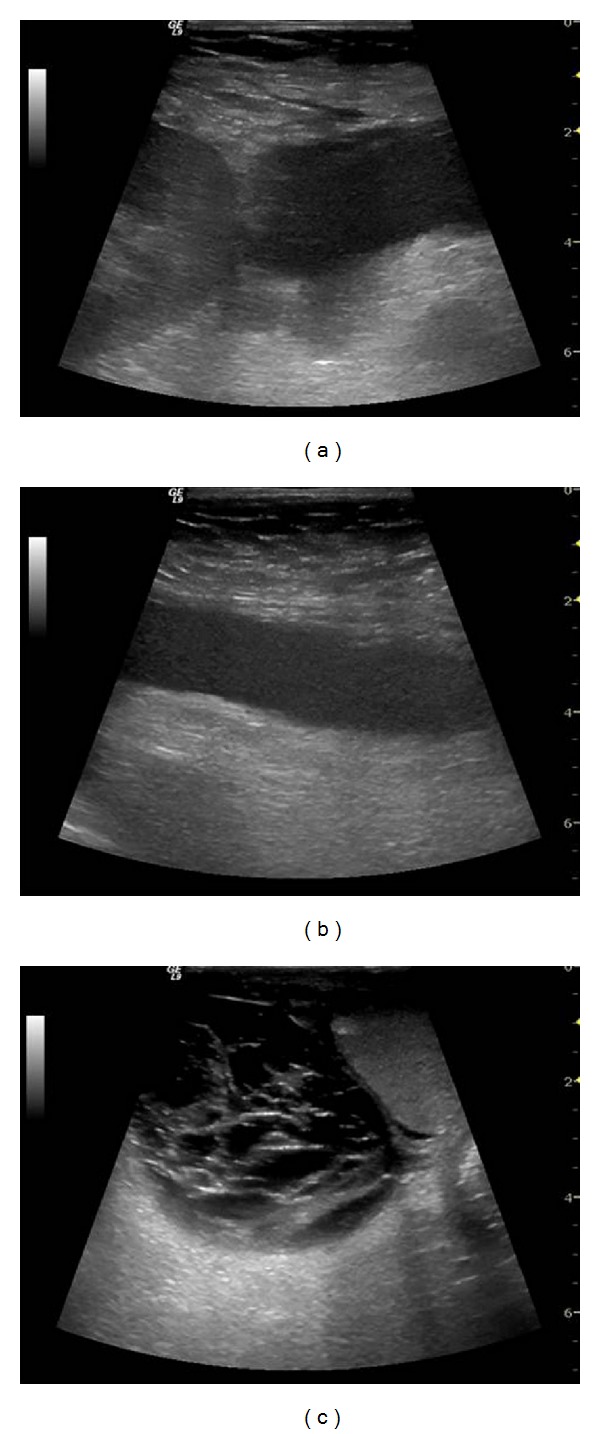
(a to c) Ultrasonographic images of a large peritransplant fluid collection 1 month after surgery, which begins from the lower part of the kidney and continues inferiorly to the scrotum. In contrast to the major part of the collection in the abdomen, which is completely anechoic, its extension in the scrotum has multiple thick septations. After ultrasonographically guided drainage the collection proved to be a lymphocele.

**Figure 15:**
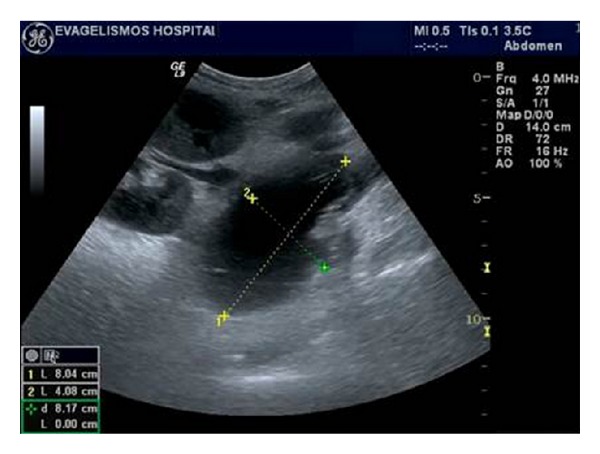
Presence of an anechoic area next to the renal transplant's hilum with no compressive effects on the graft, representing a small lymphocele on gray scale ultrasound.

On US, lymphoceles are anechoic but may contain septations and are typically positioned between the bladder and the medial aspect of the transplant (Figures 14 and 15). Although most lymphoceles are incidental findings and simply require monitoring, they have a potential to exert a mass effect on the collecting system of the transplant resulting in hydronephrosis. They may also compress the vascular pedicle of the transplant or the iliac vessels of the recipient causing edema of the lower limb, abdominal wall, scrotum, or labia [3]. Such large lymphoceles should be percutaneously or surgically drained [3, 10, 17].

#### 4.4.3. Perinephric Abscesses

Peritransplant abscesses are not observed frequently and usually develop within the first few weeks after transplantation [17]. Any perinephric collection can become infected and turn into an abscess, which is often difficult to distinguish from hematoma. Furthermore, clinical features of infection may be absent due to immunosuppression. US cannot always differentiate an abscess from other collections. The typical image of a fluid collection with low-level echoes and a thick irregular wall is very rarely found. However, if gas is seen, an abscess is probable. Power or color-Doppler may additionally illustrate increased vascularity of the wall and the surrounding tissues [40]. Contrast-enhanced ultrasound can certainly provide a more precise diagnosis. To conclude, in the pyrexial patient, any perinephric collection should be considered infected until proven otherwise through the appropriate imaging and guided diagnostic aspiration. Ultrasonography can be an effective modality to guide percutaneous drainage [10].

### 4.5. Neoplasms

Unfortunately, renal transplantation due to long-term immunosuppression places the patient at an increased risk for developing cancer (about 100 times more than general population), with a reported prevalence of 6% [41]. Although this concerns most solid tumors, the commonest seen are skin cancers and lymphomas.

The prevalence of renal adenocarcinoma may be increased, with 90% of the tumors occurring in the native kidney and 10% occurring in the transplanted kidney. Renal cell carcinomas in the allograft kidney may be introduced incidentally by the transplanted organ or develop de novo. However, they are generally less aggressive than those in the native kidneys. One reason for the increased risk of renal adenocarcinoma is that approximately half of the patients who undergo hemodialysis because of chronic renal failure develop acquired renal cystic disease. Although the cysts initially appear simple, they are dysplastic with an approximately 1% risk of malignant change [42]. Urothelial malignancies may also be seen, especially in patients with a significant exposure to cyclophosphamide (a previous generation immunosuppressive agent).

CT provides more complete information of the transplanted and the native kidneys, especially when the latter have an abnormal, shrunken appearance. However, sometimes US can be helpful identifying a solid heterogeneous enhancing mass with cystic components [17]. Nephron-sparing surgery is currently the treatment of choice. The use of percutaneous ablative techniques is yet in experimental level [10]. Lymphomas occur in approximately 1% of renal allografts and are associated with Epstein Barr virus. They may present aggressive atypical features. Posttransplantation lymphoproliferative disorder (PLTD) complicates 8% of transplantations and is diagnosed at a median of 80 months after surgery [43]. PLTD lesions are polymorphic collections of B cells indistinguishable from non-Hodgkin lymphoma. As with any lymphoma, this disorder most commonly manifests with lymphadenopathy. However, any solid organ or hollow viscera can also be affected. On ultrasonography, PLTD of the renal graft may appear as low or mixed reflective masses and tends to have a predilection for the renal hilum. Management includes cessation of immunosuppression, antiviral therapy, radiotherapy, and chemotherapy [10].

### 4.6. Recurrent Renal Disease

Advances in long-term graft survival increase the chance of recurrence of primary glomerulonephritis or secondary involvement from a systemic disease. This usually is the case in patients suffering from diabetes, amyloidosis, and cystinosis. Also, transplant recipients with active vasculitis and oxalosis are at high risk of early recurrent renal damage [44]. Ultrasonographic findings have no specific role in this task, except from excluding the treatable causes of renal dysfunction.

## 5. US Contrast Agents

The microbubble contrast agents seem to be ideal for the study of renal transplants because of their relative superficial location, fixed position, and limited respiratory movement [45]. Ultrasonography with administration of a contrast medium is an excellent and easy to perform procedure that is superior to conventional techniques such as B-mode US for volume measurement and duplex scanning for RI determination [46].

Being nonnephrotoxic, US contrast agents are safe to use in transplant kidneys, to assess focal or generalized impaired blood flow, facilitating thus the detection of ischemic lesions and AVFs [47, 48] (Figure 6). They can also be used to locate poorly perfused parenchymal renal areas, suggestive of acute pyelonephritis [49] and diagnose acute kidney graft rejection in its early stages, by identifying delayed parenchymal perfusion or perfusion defects. Furthermore, contrast-enhanced US plays a vital role in the assessment of postoperative transplant perfusion, which can be very difficult to assess accurately with conventional imaging [45].

Another indication for contrast enhanced US is in patients with perirenal hematomas, where they enable better evaluation of the extent of the collection [50]. In addition, it may also have a role in the monitoring of antirejection therapy. However, further research is required before contrast- enhanced US becomes accepted as part of the standard imaging protocol for renal allograft imaging.

## 6. Limitations

Conventional US techniques such as determination of RI and evaluation of perfusion by power Doppler have been used for years as the main tools for the diagnostic evaluation of kidney recipients in the followup of both acute and chronic rejection [51]. Nevertheless, there are limitations to the method. The examiner dependence of the US examination and the limited accessibility by Doppler US of obese patients or when the kidney lies deep within the iliac fossa frequently impairs the evaluation or leads to misinterpretation. Furthermore, the RI index is unspecific and is influenced by many factors; some of which are not related to the disease. These include the site at which the RI is measured, the increased intraabdominal pressure during forced inspiration, the pulse rate [52], or simply the immunosuppressive medication such as ciclosporin [51]. Although technical developments in US equipment and the introduction of US contrast agents have revolutionized US in recent years, renal biopsy continues to be the gold standard for diagnosing rejection in kidney recipients [46].

## 7. Summary

Kidney transplant followup is common in radiology and sonography practice. Examiners should be familiar with the anatomy, pathophysiology, and imaging findings in order to identify healthy transplants or recognize complications. Ultrasonography is the most widely applied imaging modality for kidney transplant followup as it facilitates prompt and accurate diagnosis, guiding thus treatment.

## References

[1] Vollmer WM, Wahl PW, Blagg CR (1983). Survival with dialysis and transplantation in patients with end-stage renal disease. *The New England Journal of Medicine*.

[2] Cecka JM, Terasaki PI (1992). The UNOS scientific renal transplant registry. *Clinical Transplants*.

[3] Brown ED, Chen MYM, Wolfman NT, Ott DJ, Watson NE (2000). Complications of renal transplantation: evaluation with US and radionuclide imaging. *Radiographics*.

[4] Cosgrove DO, Chan KE (2008). Renal transplants: what ultrasound can and cannot do. *Ultrasound Quarterly*.

[5] Park SB, Kim JK, Cho KS (2007). Complications of renal transplantation: ultrasonographic evaluation. *Journal of Ultrasound in Medicine*.

[6] Lachance SL, Adamson D, Barry JM (1988). Ultrasonically determined kidney transplant hypertrophy. *Journal of Urology*.

[7] Thalhammer C, Aschwanden M, Mayr M, Koller M, Steiger J, Jaeger KA (2006). Duplex sonography after living donor kidney transplantation: new insights in the early postoperative phase. *Ultraschall in der Medizin*.

[8] Irshad A, Ackerman SJ, Campbell AS, Anis M (2009). An overview of renal transplantation: current practice and use of ultrasound. *Seminars in Ultrasound, CT and MRI*.

[9] Boschiero LB, Saggin P, Galante O (1992). Renal needle biopsy of the transplant kidney: vascular and urologic complications. *Urologia Internationalis*.

[10] Parthipun A, Pilcher J (2010). Renal transplant assessment: sonographic imaging. *Ultrasound Clinics*.

[11] Irshad A, Ackerman S, Sosnouski D, Anis M, Chavin K, Baliga P (2008). A review of sonographic evaluation of renal transplant complications. *Current Problems in Diagnostic Radiology*.

[12] Lockhart ME, Wells CG, Morgan DE, Fineberg NS, Robbin ML (2008). Reversed diastolic flow in the renal transplant: perioperative implications versus transplants older than 1 month. *American Journal of Roentgenology*.

[13] Rumack CM, Wilson SF, Charboneau JW (2005). *Diagnostic Ultrasound*.

[14] Pirsch JD, Ploeg RJ, Gange S (1996). Determinants of graft survival after renal transplantation. *Transplantation*.

[15] Isoniemi HM, Krogerus L, Von Willebrand E, Taskinen E, Ahonen J, Hayry P (1992). Histopathological findings in well-functioning, long-term renal allografts. *Kidney International*.

[16] Kumar D (2010). Emerging viruses in transplantation. *Current Opinion in Infectious Diseases*.

[17] Akbar SA, Jafri SZH, Amendola MA, Madrazo BL, Salem R, Bis KG (2005). Complications of renal transplantation. *Radiographics*.

[18] Kamath NS, John GT, Neelakantan N, Kirubakaran MG, Jacob CK (2006). Acute graft pyelonephritis following renal transplantation. *Transplant Infectious Disease*.

[19] Daly BD, Goldberg PA, Krebs TL, Wong-You-Cheong JJ, Drachenberg CI (1997). End stage renal transplant failure: allograft appearances on CT. *Brain and Language*.

[20] Dodd GD, Tublin ME, Shah A, Zajko AB (1991). Imaging of vascular complications associated with renal transplants. *American Journal of Roentgenology*.

[21] Hohnke C, Abendroth D, Schleibner S, Land W (1987). Vascular complications in 1200 kidney transplantations. *Transplantation Proceedings*.

[22] Taylor KJW, Morse SS, Rigsby CM (1987). Vascular complications in renal allografts: detection with duplex Doppler US. *Radiology*.

[23] Jordan ML, Cook GT, Cardella CJ (1982). Ten years of experience with vascular complications in renal transplantation. *Journal of Urology*.

[24] Tublin ME, Dodd GD (1995). Sonography of renal transplantation. *Radiologic Clinics of North America*.

[25] Snider JF, Hunter DW, Moradian GP, Castaneda-Zuniga WR, Letourneau JG (1989). Transplant renal artery stenosis: evaluation with duplex sonography. *Radiology*.

[26] Kim SH, Kim SH (2003). Vascular diseases of the kidney. *Radiology Illustrated*.

[27] De Morais RH, Muglia VF, Mamere AE (2003). Duplex Doppler sonography of transplant renal artery stenosis. *Journal of Clinical Ultrasound*.

[28] Beecroft JR, Rajan DK, Clark TWI, Robinette M, Stavropoulos SW (2004). Transplant renal artery stenosis: outcome after percutaneous intervention. *Journal of Vascular and Interventional Radiology*.

[29] Rouviere O, Berger P, Beziat C (2002). Acute thrombosis of renal transplant artery: graft salvage by means of intra-arterial fibrinolysis. *Transplantation*.

[30] Jordan ML, Cook GT, Cardella CJ (1982). Ten years of experience with vascular complications in renal transplantation. *Journal of Urology*.

[31] Reuther G, Wanjura D, Bauer H (1989). Acute renal vein thrombosis in renal allografts: detection with duplex Doppler US. *Radiology*.

[32] Surlan M, Popovic P (2003). The role of interventional radiology in management of patients with end-stage renal disease. *European Journal of Radiologyl*.

[33] Brandenburg VM, Frank RD, Riehl J (2002). Color-coded duplex sonography study of arteriovenous fistulae and pseudoaneurysms complicating percutaneous renal allograft biopsy. *Clinical Nephrology*.

[34] Donckier V, De Pauw L, Ferreira J (1995). False aneurysm after transplant nephrectomy: report of two cases. *Transplantation*.

[35] Koçak T, Nane I, Ander H, Ziylan O, Oktar T, Ozsoy C (2004). Urological and surgical complications in 362 consecutive living related donor kidney transplantations. *Urologia Internationalis*.

[36] Bennett LN, Voegeli DR, Crummy AB (1986). Urologic complications following renal transplantation: role of interventional radiologic procedures. *Radiology*.

[37] Yong AA, Ball ST, Pelling MX, Gedroyc WM, Morgan RA (1999). Management of ureteral strictures in renal transplants by antegrade balloon dilatation and temporary internal stenting. *CardioVascular and Interventional Radiology*.

[38] De Francisco AM, Riancho JA, Amado JA (1987). Calcium, hyperparathyroidism, and vitamin D metabolism after kidney transplantation. *Transplantation Proceedings*.

[39] Pozniak MA, Dodd GD, Kelcz F (1992). Ultrasonographic evaluation of renal transplantation. *Radiologic Clinics of North America*.

[40] Baxter GM (2003). Imaging in Renal Transplantation. *Ultrasound Quarterly*.

[41] Gallagher MP, Kelly PJ, Jardine M (2010). Long-term cancer risk of immunosuppressive regimens after kidney transplantation. *Journal of the American Society of Nephrology*.

[42] Schwarz A, Vatandaslar S, Merkel S, Haller H (2007). Renal cell carcinoma in transplant recipients with acquired cystic kidney disease. *Clinical Journal of the American Society of Nephrology*.

[43] Vrachliotis TG, Vaswani KK, Davies EA, Elkhammas EA, Bennett WF, Bova JG (2000). CT findings in posttransplantation lymphoproliferative disorder of renal transplants. *American Journal of Roentgenology*.

[44] Matthew TH (1991). Recurrent disease after transplantation. *Transplantation Reviews*.

[45] McArthur C, Baxter GM (2012). Current and potential renal applications of contrast-enhanced ultrasound. *Clinical Radiology*.

[46] Fischer T, Filimonow S, Dieckhöfer J (2006). Improved diagnosis of early kidney allograft dysfunction by ultrasound with echo enhancer—a new method for the diagnosis of renal perfusion. *Nephrology Dialysis Transplantation*.

[47] Helck A, Sommer WH, Wessely M (2011). Benefit of contrast enhanced ultrasound for detection of ischaemic lesions and arterio venous fistulas in renal transplants—a feasibility study. *Clinical Hemorheology and Microcirculation*.

[48] Grzelak P, Sapieha M, Kurnatowska I, Nowicki M, Strzelczyk J, Stefańczyk L (2011). Contrast-enhanced sonography of postbiopsy arteriovenous fistulas in kidney grafts. *Journal of Clinical Ultrasound*.

[49] Granata A, Andrulli S, Fiorini F (2011). Diagnosis of acute pyelonephritis by contrast-enhanced ultrasonography in kidney transplant patients. *Nephrology Dialysis Transplantation*.

[50] Fischer T, Dieckhofer J, Lembcke A (2005). The use of contrast-enhanced US in renal transplant: first results and potential clinical benefit. *European Radiology*.

[51] Radermacher J, Mengel M, Ellis S (2003). The renal arterial resistance index and renal allograft survival. *The New England Journal of Medicine*.

[52] Takano R, Ando Y, Taniguchi N, Itoh K, Asano Y (2001). Power Doppler sonography of the kidney: effect of Valsalva’s maneuver. *Journal of Clinical Ultrasound*.

